# Comparison of the Tendon Damage Caused by Four Different Anchor Systems Used in Transtendon Rotator Cuff Repair

**DOI:** 10.1155/2012/798521

**Published:** 2012-07-01

**Authors:** Qing-Song Zhang, Sen Liu, Qiuyang Zhang, Yun Xue, Dongxia Ge, Michael J. O'Brien, Felix H. Savoie, Zongbing You

**Affiliations:** ^1^Department of Structural and Cellular Biology, and Tulane Cancer Center, LCRC, Tulane Center for Aging, Tulane Center for Stem Cell Research and Regenerative Medicine, Tulane University School of Medicine, New Orleans, LA 70112, USA; ^2^Department of Orthopaedic Surgery and Tulane Institute of Sports Medicine, Tulane University School of Medicine, New Orleans, LA 70112, USA; ^3^Department of Orthopaedic Surgery, Pu Ai Hospital of Tongji Medical College, Huazhong University of Science and Technology, Wuhan 430033, China

## Abstract

*Objectives*. The objective of this study was to compare the damage to the rotator cuff tendons caused by four different anchor systems. *Methods*. 20 cadaveric human shoulder joints were used for transtendon insertion of four anchor systems. The Healix Peek, Fastin RC, Bio-Corkscrew Suture, and Healix Transtend anchors were inserted through the tendons using standard transtendon procedures. The areas of tendon damage were measured. *Results*. The areas of tendon damage (mean ± standard deviation, *n* = 7) were 29.1 ± 4.3 mm^2^ for the Healix Peek anchor, 20.4 ± 2.3 mm^2^ for the Fastin RC anchor, 23.4 ± 1.2 mm^2^ for the Bio-Corkscrew Suture anchor, 13.7 ± 3.2 mm^2^ for the Healix Transtend anchor inserted directly, and 9.1 ± 2.1 mm^2^ for the Healix Transtend anchor inserted through the Percannula system (*P* < 0.001 or *P* < 0.001, compared to other anchors). *Conclusions*. In a cadaver transtendon rotator cuff repair model, smaller anchors caused less damage to the tendon tissues. The Healix Transtend implant system caused the least damage to the tendon tissues. Our findings suggest that smaller anchors should be considered when performing transtendon procedures to repair partial rotator cuff tears.

## 1. Introduction

The rotator cuff of the shoulder joint is made up of four muscles (the supraspinatus, infraspinatus, subscapularis, and teres minor) and their tendons. Its function is to lift and rotate the arm and to stabilize the humeral head against the glenoid. Partial-thickness rotator cuff tears (PRCTs) are a common component of rotator cuff disease, bridging the gap from tendinitis to frank tendon disruption. PRCT has been divided into three types based on its location, including bursal surface, articular surface, and intratendinous tears [1, 2]. Histological studies have shown that anatomical differences make the articular portion of the rotator cuff tendons more vulnerable to tension [3–5]. Snyder and colleagues used the term PASTA to represent partial articular supraspinatus tendon avulsion [6, 7]. PASTA has a high likelihood of progression to complete tears in patients who are not repaired timely, leading to consistent pain and disability of the shoulder [6, 7].

Arthroscopic surgery is indicated in the patients whose PRCT has failed conservative treatments [8]. Two common surgical treatments include debridement of the tear with or without acromioplasty, or converting the PRCT into a complete tear and then repairing the tendon onto the humeral footprint. The disadvantage of this second treatment option is that the intact portion of the tendon is cut, and it may potentially create a length-tension mismatch in the repaired rotator cuff [9, 10]. In order to preserve the intact portion of the tendon, the transtendon repair technique has been developed [10]. In the transtendon procedure, a small perforation is made either with a self-tapping anchor (and then the anchor is inserted into the underlying bone), or with an awl/tap instrument followed by anchor insertion through the tendon and into the underlying bone. The suture attached to the anchor is used to repair the torn portion of the tendon. It has been reported that PASTA lesions greater than 50% of the width of the rotator cuff tendon are best managed with surgical repair [11–13]. Currently, transtendon repair has become more and more popular, because this technique allows the surgeon to selectively repair the torn portion of the tendon while minimizing any length-tension mismatch of the repaired rotator cuff muscles [14–17]. The disadvantage of the transtendon procedure is that the tendon at the perforated site is damaged. Lyons et al. reported an alternative method to repair PASTA, in which the tear was closed by a side-to-side suture of the supraspinatus tendon to subscapularis tendon, thus placing the debrided tendon against the bony footprint without an anchor [18]. However, most surgeons prefer repairing the tendon to bone by suture anchors, in order to obtain an anatomical restoration of the rotator cuff [10].

The adverse effects of the tendon damage with a transtendon repair on the healing and mechanical strength of the repaired tendon are unclear. However, it is reasonable to believe that less damage to the tendon causes less adverse effects. Our hypothesis is that anchors with a smaller diameter may cause less damage to the tendon. The purpose of this study was to compare the tendon damage caused by four different anchor systems, in order to guide our choice of anchor systems in the transtendon procedure.

## 2. Methods

### 2.1. Source of Specimens and Anchors

This study was performed on cadaveric shoulder joints. Twenty unembalmed human shoulder specimens were obtained from donors through the Bureau of Anatomical Services, Louisiana State Department of Health and Hospitals. The donor ages were from 54 to 88 (average 75.9) years old. The use of these deidentified specimens was determined as “not human subjects study” by the Tulane University Institutional Review Board (Project no. 206610-1). The specimens were stored at −20°C and thawed at room temperature prior to use. Three anchor systems were manufactured by Depuy Mitek, Inc. (Raynham, MA), including the Healix Peek anchor (5.5 mm in diameter), Fastin RC anchor (5.0 mm in diameter), and the Healix Transtend implant system which consists of a Healix Transtend Peek anchor (3.4 mm in diameter) and the Percannula system (a percutaneous cannula with a 4.0 mm diameter). The Bio-Corkscrew Suture anchor (5.0 mm in diameter) was manufactured by Arthrex, Inc. (Naples, FL).

### 2.2. Transtendon Surgical Procedure

**Figure 1:**
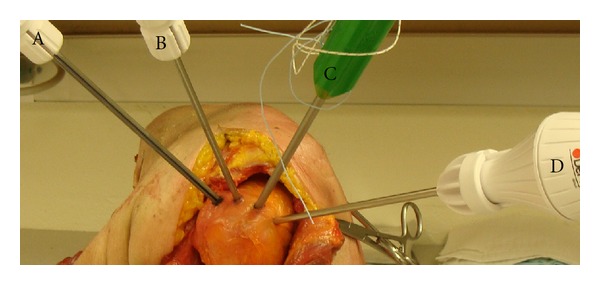
Transtendon insertion of the anchors. The supraspinatus and infraspinatus tendons were exposed by a superior-lateral approach, after dissecting the skin and subcutaneous tissue and retraction of the anterior portion and middle portion of the deltoid muscle. Four anchors were inserted into the humeral footprint, including the Healix Peek (A), Fastin RC (B), Bio-Corkscrew Suture anchor (C), and Healix Transtend (D).

Each specimen was mounted on a custom apparatus. The supraspinatus and infraspinatus tendons of each specimen were exposed after dissecting the skin and subcutaneous tissue and retraction of the deltoid muscle (Figure 1). The rotator cuff footprint on the proximal humerus was identified. Next, an awl or tap was used to perforate the tendon and the underlying bone for the Healix Peek anchor, the Bio-Corkscrew Suture anchor, and the Healix Transtend anchor insertions. Since the Fastin RC anchor is a self-tapping anchor, no awl or tap is required to prepare a hole in the bone. The next step was to insert the anchors. The anchors were inserted into the footprint area at a 45° angle (dead man's angle) to the direction of contractile force of the rotator cuff muscles, in order to mimic the clinical practice. Then, a second anchor was similarly inserted at a distance of ≥5 mm to the first anchor. In order to compare the tendon damages side by side, the 4 anchors were inserted into the same joint in two specimens (Figure 1). In the remaining specimens, two anchors were inserted. The Healix Peek, Fastin RC, and Bio-Corkscrew Suture anchors were inserted directly through the tendons into the underlying rotator cuff footprint (not through a transtendon cannula, as there are no cannula components in these anchor implant systems). The Healix Transtend implant system includes a percutaneous cannula (the Percannula system, 4.0 mm in diameter), thus the Healix Transtend anchor was inserted either without any cannula (in order to compare it with other anchors under similar insertion conditions) or through the Percannula system (in order to mimic its clinical usage). The procedure to insert the Percannula system followed the manufacturer's instructions. The cannula was first positioned onto the footprint. Then, the awl/tap was inserted through the cannula and was used to create the bone hole. Next, the awl/tap was removed and the anchor was inserted through the cannula and into the bone. Black pen oil was painted on the outer surfaces of anchors or cannulae, so as to provide a contrast color for taking photographs of the tendon damage.

### 2.3. Measurement of Tendon Damage

After removing the sutures from the anchors (of note, the sutures were not tied because the purpose was to compare tendon damage caused by anchors), the tendon damage was clearly marked by the black pen oil. Photographs of the tendon damage were taken together with a ruler as a standard of size and transformed into tagged image file format (TIFF) files to fit the computer software used for image analysis. The areas of tendon damage on the photographs were traced along the margin of the black marks and then measured automatically by computer software (Quantity One version 4.6.5, Bio-Rad Laboratories, Hercules, CA). The measured areas were calibrated by the ruler included in each photograph.

### 2.4. Visualization of Microscopic Tendon Damage

The impacted tendons were embedded in the optimal cutting temperature (OCT) compound and cut into 200 *μ*m thick frozen sections. The sections were scanned with a confocal microscope (Leica TCS SP2, Leica Microsystems, Exton, PA). Ten optical sections were scanned per tendon sample with a magnification of 100x. Each section was 5 *μ*m apart starting approximately 50 *μ*m away from the cutting surface, in order to avoid the sectioning artifacts. The ten optical sections were stacked into one picture.

### 2.5. Statistical Analysis

The areas of tendon damage were presented as the mean and standard deviation of 7 anchors per each type of anchor system and were analyzed with two tailed Student's *t*-test. The level of significance was set at *P* < 0.05.

## 3. Results

**Figure 2:**
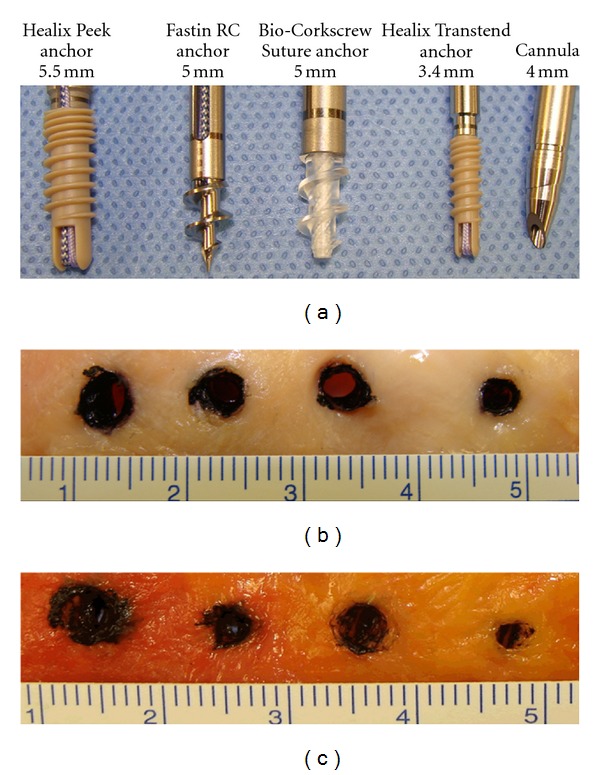
Photographs of anchors and the tendon damages caused by the anchors. (a) Photographs of four types of anchors studied, that is, the Healix Peek, Fastin RC, Bio-Corkscrew Suture, and Healix Transtend (with the Percannula system) anchors. (b) Photographs of tendon damage caused by the four types of anchors. All anchors were inserted through the supraspinatus and infraspinatus tendons without any cannula. (c) Photographs of tendon damage caused by the four types of anchors. The Healix Peek, Fastin RC, and Bio-Corkscrew Suture anchors were inserted through the supraspinatus and infraspinatus tendons without any cannula. The Healix Transtend anchor was inserted through the Percannula system, thus the hole in the tendon was caused by the cannula. The unit of the rulers was cm.

**Figure 3:**
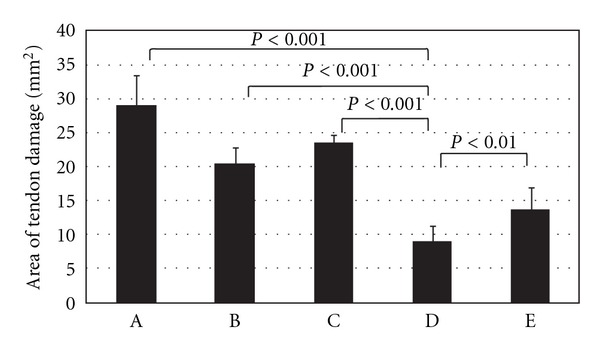
The areas of tendon damage caused by the anchors. A. The Healix Peek anchor; B. The Fastin RC anchor; C. The Bio-Corkscrew Suture anchor; D. The Healix Transtend anchor inserted through the Percannula system; and E. The Healix Transtend anchor inserted without any cannula.

We found that all of the four types of anchors (Figure 2(a)) made a hole-like area of damage when they were inserted through the tendon (Figures 2(b) and 2(c)). As shown in Table 1 and Figure 3, the areas of tendon damage (mean ± standard deviation, *n* = 7) were 29.1 ± 4.3 mm^2^ for the Healix Peek anchor, 20.4 ± 2.3 mm^2^ for the Fastin RC anchor, 23.4 ± 1.2 mm^2^ for the Bio-Corkscrew Suture anchor, 9.1 ± 2.1 mm^2^ for the Healix Transtend anchor inserted through the Percannula system, and 13.7 ± 3.2 mm^2^ for the Healix Transtend anchor inserted without any cannula. The differences of the areas were statistically significant between any two types of anchors (*P* < 0.01 or 0.001) (Table 1). The area of tendon damage caused by the Healix Transtend anchor (i.e., when it was inserted without the cannula) was significantly larger than that caused by the Percannula system (i.e., when the anchor was inserted within the cannula) (*P* < 0.01).

**Figure 4:**
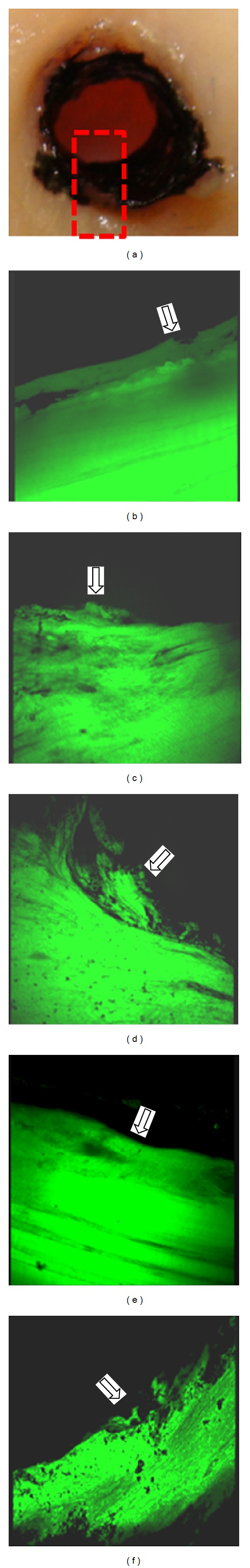
Microscopic structures of the damaged tendon tissues. (a) The rectangular area illustrated where the tendon tissues were sampled for frozen sectioning and confocal microscopy. (b) to (f) Representative microscopic structures of the tendons damaged by the Healix Peek anchor (b), the Fastin RC anchor (c), the Bio-Corkscrew Suture anchor (d), the Healix Transtend anchor inserted through the Percannula system (e), and the Healix Transtend anchor inserted without any cannula (f). Orientation of the photographs: top, edge of the damaged tendon (arrows); bottom, toward the normal part of the tendon. The green fluorescence was the autofluorescence of the tendon fibers. Original magnification, 100x.

The impacted tendon tissues were sampled as shown in Figure 4(a) for examination of microscopic structures. Under the microscope, the tendons showed signs of fragmentation of tendon fibers when they were impacted by the Healix Peek, Fastin RC, and Bio-Corkscrew Suture anchors (Figure 4(b) to 4(d)). In contrast, the tendons that were impacted by the Percannula system of the Healix Transtend implant system showed signs of slight compression with no fragmentation of the tendon fibers (Figure 4(e)). Without the use of the cannula, the Healix Transtend anchor also fragmented the tendon fibers at the edge of the impacted tendon (Figure 4(f)).

## 4. Discussion

The present study found that the anchor with the largest diameter (i.e., the Healix Peek anchor, 5.5 mm in diameter) made the biggest area of tendon damage (29.1 mm^2^) and the anchor with the smallest diameter (i.e., the Healix Transtend anchor, 3.4 mm in diameter) produced the smallest area of tendon damage (13.7 mm^2^) under similar insertion conditions. The Fastin RC and Bio-Corkscrew Suture anchors with the intermediate diameter (5.0 mm) caused intermediate tendon damage (i.e., 20.4 mm^2^ and 23.4 mm^2^, resp.). These findings support our hypothesis that the anchor with a smaller diameter may cause less damage to the tendon, which is logical and predictable. Surprisingly, both the Fastin RC anchor and Bio-Corkscrew Suture anchor have a diameter of 5.0 mm, yet the area of tendon damage caused by the Fastin RC was significantly smaller than the area of tendon damage caused by the Bio-Corkscrew Suture anchor (*P* < 0.01). We speculate that the difference may be due to the different material and shape of the anchor. The Fastin RC anchor is made of titanium alloy, whereas the Bio-Corkscrew Suture anchor is made of bioabsorbable poly-L/D-lactide copolymer. It is possible that the metal material has less friction than the polymer, hence the tendon tissue is more likely to be pushed outward by the metal anchor, rather than being trapped and crushed under the threads. The threads of the Fastin RC anchor are thinner and face more downward than the Bio-Corkscrew Suture anchor, thus making the Fastin RC anchor, at least the anchor's core cylinder, appear smaller than the Bio-Corkscrew Suture anchor (Figure 2(a)). Also surprising, although the 4.0 mm diameter of the Percannula system is larger than the 3.4 mm diameter of the Healix Transtend anchor, the area of tendon damage (9.1 mm^2^) caused by the Percannula system was significantly smaller than that caused by the anchor (13.7 mm^2^) (*P* < 0.01). We suspect that, because the metal cannula has a smooth surface and tapered tip (of note, the tip is solid when the system's obturator is placed inside the cannula), the tendon tissue was pushed outward when the cannula was inserted, rather than being screwed and crushed by the anchor that is made of polyetherether ketone material and with threads. When the cannula was removed, the tendon tissues partially rebounded, thus leaving a hole that was smaller than the cannula's diameter. We predict that, in clinical practice, the live tendon tissues may have much better flexibility than the cadaveric tendon tissues, so that the tendon tissue may rebound more and leave a much smaller hole. This interpretation is supported by our microscopic findings that all of the anchors fragmented the tendon fibers (Figure 4(b), 4(c), 4(d), and 4(f)). The signs of fragmented fibers in addition to lack of fibers in the holes suggest that the tendon fibers are likely transected by the anchors, at least in the center of tendon damage. In contrast, the Healix Transtend implant system (Healix Transtend anchor inserted through the Percannula system) did not fragment the tendon fibers (Figure 4(e)). Instead, the cannula-impacted tendon fibers showed signs of compression (Figure 4(e)). Since the cannula appears to reduce the tendon damage (see Table 1, comparing the Healix Transtend anchor with or without the cannula), it is reasonable to speculate that the mechanical crushing injury to the tendon may be mitigated by using an #11 blade scalpel to cut a small incision in the tendon prior to insertion of the anchors.

We have provided evidence showing smaller anchors cause less damage. One logical question to ask is whether the smaller anchors provide adequate fixation strength. Data released by DePuy Mitek showed that the average load to failure is 67 pounds (298 Newtons) for the 5.5 mm Healix Peek anchor, 51 pounds (227 Newtons) for the 5.5 mm Bio-Corkscrew FT anchor, and 49.6 pounds (221 Newtons) for the 3.4 mm Healix Transtend Peek anchor. Thus, the small anchor only has slightly less fixation strength compared to the large anchors. It is worth pointing out that the Healix Transtend Peek anchor is recommended to use in duplex. Under such circumstances, the combined area of tendon damage caused by two Healix Transtend anchors (inserted with the Percannula system) is still smaller than any of the large anchors used singly.

The limitation of this study was the usage of cadaveric specimens. The cadaveric tendon tissues may have less flexibility than live tendons in human patients. Therefore, the damage caused by the anchors could be greater than in live tendons due to their limited flexibility. The second limitation was that we did open surgery while the anchors are mainly made for arthroscopic surgery. We believe that open surgery simplified our procedure and avoided some confounding factors, such as false passes and incorrect locations that might be caused by the complexity of the arthroscopic surgery. In addition, because we need to use oil marks to show the tendon damage for accurate measurement, passing anchors through skin and muscles in an arthroscopic surgery would remove our marks. Therefore, we believe that open surgery is appropriate for the purpose of this study. The third limitation is that, by using cadaveric specimens, it is not possible to evaluate the effects of the size of the tendon damage on tendon healing. It is possible that the damaged tendon could be repaired after the surgery, regardless of the size of the damage. As a general principle, less damage is preferred in surgery. Thus, animal study is warranted to compare the different anchors used in transtendon repair.

## 5. Conclusion

In a cadaver transtendon rotator cuff repair model, smaller anchors caused less damage to the tendon tissues. The Healix Transtend implant system (consisting of the Healix Transtend anchor and the Percannula system) among the anchors tested caused the least damage to the tendon tissues. Our findings suggest that smaller anchors should be considered when performing transtendon procedures to repair partial rotator cuff tears.

## Figures and Tables

**Table 1:** Areas of tendon damage caused by anchors.

Sample number	(a) Healix Peek anchor (5.5 mm)	(b) Fastin RC anchor (5.0 mm)	(c) Bio-Corkscrew Suture anchor (5.0 mm)	(d) Healix Transtend anchor inserted through the Percannula system (4.0 mm)	(e) Healix Transtend anchor (3.4 mm) without cannula
1	25.0	17.4	23.3	7.0	13.0
2	28.6	22.6	21.4	10.7	10.0
3	31.6	22.8	22.9	9.1	10.1
4	37.5	18.6	24.7	10.9	16.7
5	25.8	22.7	22.9	6.6	18.7
6	26.8	18.3	23.7	7.5	14.1
7	28.5	20.2	24.8	11.7	13.1
Mean	**29.1**	**20.4**	**23.4**	**9.1**	**13.7**
SD	**4.3**	**2.3**	**1.2**	**2.1**	**3.2**
*P* (versus a)		0.0005	0.0052	0.0000	0.0000
*P* (versus b)			0.0099	0.0000	0.0008
*P* (versus c)				0.0000	0.0000
*P* (versus d)					0.0078

Note: SD represents standard deviation. *P* values were obtained by the two-tailed Student's *t*-test, comparing between two types of anchors as indicated.
